# Systemic Therapy for Non-Melanoma Skin Cancers: Latest Advances

**DOI:** 10.1007/s11912-024-01570-1

**Published:** 2024-07-02

**Authors:** Spencer Lessans, Katie A. O’Connell, Jennifer Choe

**Affiliations:** 1 Department of Internal Medicine, Vanderbilt University Medical Center, Nashville, TN USA; 2 Department of Dermatology, Vanderbilt University Medical Center, Nashville, TN USA; 3 Department of Hematology/Oncology, Vanderbilt University Medical Center, Preston Research Building 790, 2220 Pierce Ave, Nashville, TN 37232 USA

**Keywords:** Squamous cell carcinoma, Basal cell carcinoma, Merkel cell carcinoma, Immunotherapy, Neoadjuvant

## Abstract

**Purpose of Review:**

This review provides an update on approved and emerging systemic therapies in the treatment of locally advanced or metastatic non-melanoma skin cancers (squamous cell carcinoma, basal cell carcinoma, Merkel cell carcinoma).

**Recent Findings:**

Many studies demonstrate the effectiveness of immunotherapy for all types of non-melanoma skin cancer. For basal cell carcinoma (BCC), hedgehog inhibitors (HHI) remain first-line but with poor tolerability. Numerous clinical trials studying both neoadjuvant and adjuvant use of anti-PD-1 and anti-PD-L1 therapies in advanced NMSC are under investigation.

**Summary:**

There is a growing number of systemic therapies available to treat non-melanoma skin cancers. The advent of immunotherapy has revolutionized the field and greatly improved survival compared to historical survival rates with cytotoxic chemotherapy.

## Introduction

Non-melanoma skin cancer (NMSC) is a term used to describe a broad range of skin malignancies. It most commonly includes keratinocyte carcinomas: cutaneous squamous cell carcinoma (CSCC) and basal cell carcinoma (BCC), but also includes Merkel cell carcinomas (MCC). There are approximately 5.4 million cases of NMSC each year, 80% of which occur in people aged 60 years or older [1, 2]. NMSC is the most common cancer type by incidence and, while it has a low mortality rate, the total number of mortalities is close to that of melanoma due to its high prevalence [3]. The incidence of both BCC and SCC have steadily increased [4]. This is thought to be due, at least in part, to the cumulative exposure to ultraviolet (UV) radiation, which is associated with 90% of NMSC cases [5].

Surgical resection utilizing peripheral and deep margin assessment (PDEMA) which includes Mohs surgery and surgical excision using standard vertical sectioning remain the mainstay of treatment for the majority of cases amenable to resection [6]. For cases not amenable to surgical resection, cytotoxic chemotherapy had historically been utilized. Studies looking at outcomes for patients with advanced or metastatic BCC or CSSC were limited to case series, small sample sizes, and lack of randomization, but had limited convincing efficacy prior to the advent or targeted molecular therapies and immunotherapy [7]. In 2011, the first checkpoint inhibitor, ipilimumab, was approved for use in metastatic melanoma followed by approval of pembrolizumab [8]. Since then, immunotherapy has revolutionized cancer therapy, expanding not only into other non-melanoma skin cancers but across solid tumors. The growth of immunotherapy and targeted molecular therapy in treatment of locally advanced and metastatic NMSC has significantly changed treatment options currently available to patients and therefore survival in this population. This review summarizes systemic therapies currently available for treatment of locally advanced and metastatic non-melanoma skin cancers and highlights emerging therapies under investigation.

## Cutaneous Squamous Cell Carcinoma

CSCC is the second most common skin cancer behind BCC. Previous efforts to characterize the genetic landscape of CSCC have proven difficult given high background mutation rates as a result of ultraviolet damage [9]. UV radiation-induced mutations in TP53 play an important role early in the pathogenesis of CSCC. Mutations in NOTCH1, NOTCH2, CDKN21, HRAS, and FAT1 are also common, while mutations in KMT2C have been associated with poor outcomes and increased bone invasion. Additionally, CSCC displays one of the highest tumor mutational burden (TMB) among all solid tumors and high TMB is associated with increased response to immunotherapy [10].

Advanced CSCC includes both locally advanced CSCC (laCSCC) not amenable to surgery or radiotherapy and metastatic CSCC (mCSCC). For advanced CSCC where a cure is unlikely following surgery or radiotherapy, primary treatment options defined by NCCN CSCC Guidelines Version 1.2024 include neoadjuvant cemiplimab after multidisciplinary discussion and surgical resection utilizing PDEMA or standard excision with wider surgical margins and postoperative margin assessment. For non-surgical candidates, standard of care includes radiotherapy in combination with systemic therapy or systemic therapy alone if curative radiotherapy is not feasible. Guidelines suggest considering neoadjuvant cemiplimab for rapidly growing tumors, in-transit metastasis, lymphovascular invasion, borderline resectability, or if surgery alone may not be curative or may result in significant functional limitations. Preferred systemic regimens include cemiplimab, pembrolizumab, or a clinical trial. In patients who are ineligible for or have progressed on immunotherapy and clinical trials, carboplatin, paclitaxel, and cetuximab may be considered [11].

### Chemotherapy and Targeted Therapy

Prior to immunotherapy, both targeted therapy and chemotherapy were used for advanced CSCC with generally poor responses. Epidermal growth factor receptor (EGFR) inhibitors as monotherapy have been associated with an objective response rate (ORR) of 10–31% [12–15]. Data on chemotherapy are limited to retrospective studies or prospective studies with small sample sizes. Although immunotherapy has dramatically changed outcomes for patients, treatment in those with immunotherapy-refractory or ineligible disease still primarily relies upon chemotherapy and/or targeted therapy. In a recent retrospective analysis of patients treated with cetuximab, among 11 patients who were given cetuximab following immunotherapy failure, the ORR was 64% with quick and durable responses, suggesting cetuximab should be considered as the preferred therapy following immunotherapy failure [16].

### Immunotherapy

#### Cemiplimab

Cemiplimab is a monoclonal antibody directed against programmed death 1 (PD-1). In September 2018, the Food and Drug Administration (FDA) approved cemiplimab for treatment of mCSCC or laCSCC in patients who are not candidates for curative surgery or radiation [17]. This marked the first time that the FDA officially approved any systemic therapy for advanced CSCC, for which no standard-of-care existed. Approval was based on a phase I study which included patients with laCSCC and mCSCC as well as a phase II study of patients with mCSCC. In the phase I study, the ORR was 50% while the phase II study reported an ORR of 47% with 4 complete responses (CR) at median follow-up of 7.9 months. Notable adverse events (AE) were cellulitis, pneumonitis, hypercalcemia, pleural effusion, and death [18]. In another study of 78 patients with laCSCC given cemiplimab, the ORR was 44% where 10 patients achieved CR at median follow up of 9.3 months [19]. Another clinical trial investigated fixed-dose cemiplimab compared to weight-based dosing and found an ORR of 45.2% where patients with fixed-dose cemiplimab achieved comparable results to weight-based dosing (ORR of 41.1% vs 49.2%) [20]. EMPOWER-CSCC 1 was the largest pooled dataset of patients with advanced CSCC treated with cemiplimab and reported an ORR of 46.1% at median follow-up of 15.7 months. Additionally, improved CR were observed over time given increased CR in this study as compared with primary analyses as above. Among the 31 complete responders, median time to CR was 11.2 months [21].

#### Pembrolizumab

Pembrolizumab is another monoclonal antibody targeting PD-1 that became the second immunotherapy agent approved for CSCC in June 2020 for patients with recurrent or mCSCC not curable by surgery or radiation [22]. Later, FDA approval was expanded to include patients with laCSCC not curable by surgery or radiation. Initial approval was based upon data from KEYNOTE-629, a phase II non-randomized study of patients with recurrent or mCSCC. This study also included a cohort of patients with laCSCC. Among patients with recurrent or metastatic disease, the ORR was 35.2%. Among those who responded, 10.5% achieved CR. For patients with laCSCC, the ORR was 50% with 16.7% achieving CR [23]. 69.2% of patients experienced one or more treatment-related AEs. 14 patients discontinued therapy due to AEs and 2 patients died from fatal AEs.

CASKIN was another phase II study of pembrolizumab for advanced CSCC with an ORR of 42% with 4 patients achieving CR and median overall survival was 25.3 months [24].

The I-TACKLE trial (NCT03666325) is a nonrandomized phase 2 trial for patients with laCSCC and mCSCC with pembrolizumab. If patients achieve either a PR or CR, they continued on pembrolizumab. However, in the case of stable disease or disease progression, patients received both pembrolizumab and cetuximab. Thus far, 43 patients have been enrolled and 23 underwent combination treatment (17 PD and 6 SD). 21 patients were noted to have primary resistance and 2 had acquired resistance. Cumulative ORR was 63% with 44% responding to pembrolizumab alone and 38% responding to combination therapy given primary resistance. In the 2 patients with acquired resistance, both achieved PR following combination therapy. Therefore, a total of 44% of patients responded to combination therapy. Combination therapy resulted in one-year PFS of 42% while monotherapy had 51%. Grade ≥ 3 AEs occurred in 35% of patients on combination therapy and 16% of patients on pembrolizumab alone with 3 patients on monotherapy discontinuing treatment secondary to AEs [25].

#### Nivolumab

Nivolumab is another PD-1 inhibitor being studied for patients with advanced CSCC. A single-arm phase 2 study of nivolumab for systemic treatment-naïve patients with advanced CSCC included 24 patients (4 locally advanced, 16 locoregional, 4 metastatic). Best objective response rate of 58.3% was reported at 24 weeks (0 CR, 14 PR). The incidence of grade 3 or higher immune related adverse events (irAEs) was 25% and only one patient discontinued therapy due to toxicity [26].

Another multicenter, single-arm phase 2 study (NIVOSQUACS) reported impressive results in patients with laCSCC or mCSCC given nivolumab for up to 24 months. Notably, patients with concomitant hematological malignancies (non-progressing or stable under active therapy) were eligible for inclusion and 35% of patients had a coexisting hematological malignancy. The ORR was 61.3% and 22.6% of patients achieved an investigator-assessed complete response. The incidence of grade ≥ 3 irAEs was 19.4% and two patients discontinued nivolumab due to irAEs [27].

### Neoadjuvant Use

Neoadjuvant use of cemiplimab has been studied in a phase 2 trial with ORR of 68% in patients with stage II-IV (M0) CSSC and CR in 51%, demonstrating high therapeutic activity in patients with resectable disease [28]. Neoadjuvant therapy allowed for decreased surgical morbidity with preservation of important functional structures. Although not FDA approved since pathologic response rates are not considered a validated endpoint by the FDA, neoadjuvant cemiplimab has become commonplace due to its impressive response rates. It remains unknown if the pathologic response rate translates to improved survival outcomes to support its use as an appropriate surrogate. Despite a lack of long-term outcomes, neoadjuvant cemiplimab has been practice-changing given the potential for reduced surgical morbidity. Since half of all patients develop a complete pathologic response with neoadjuvant cemiplimab, this has resulted in questions about how to best manage sites of disease that are no longer clinically evident and whether definitive interventions of surgery or radiation are necessary. A phase 3 trial evaluating neoadjuvant cemiplimab with survival outcomes is currently under development to determine whether pathologic or clinical response translates to long-term overall survival or progression-free survival benefit.

Several neoadjuvant trials are currently underway assessing use of cemiplimab, pembrolizumab, nivolumab, and nivolumab with ipilimumab for patients with resectable CSCC (Table 1). Case series have reported success with neoadjuvant pembrolizumab in patients with advanced CSCC [29, 30]. Additionally, a trial involving neoadjuvant use of a co-formulated LAG3 inhibitor (favezelimab) plus pembrolizumab (MK-4280A) vs pembrolizumab monotherapy is ongoing (NCT06036836). Neoadjuvant atezolizumab, cetuximab, and erlotinib are also being investigated.

**Table 1:** Ongoing Clinical Trials open to patients with Cutaneous Squamous Cell Carcinoma

Setting	Agent(s)	Population	Phase	NCT identifier
ADVANCED CSCC—MONOTHERAPY				
	Pembrolizumab	laCSCC, mCSCC	II	NCT02964559
	Pembrolizumab	laCSCC, mCSCC	II	NCT02721732
	INCB099280 (small-molecule PD-L1 inhibitor)	advanced CSCC	I	NCT04242199
	INCB099280 (small-molecule PD-L1 inhibitor)	laCSCC, mCSCC	II	NCT05888844
	INCB099318 (small molecule PD-L1 inhibitor)	advanced CSCC	I	NCT04272034
	HLX07 (Recombinant Humanized Anti-EGFR Monoclonal Antibody)	laCSCC, mCSCC	II	NCT05238363
	Afatinib (tyrosine kinase inhibitor)	laCSCC, mCSCC	II	NCT05070403
ADVANCED CSCC—COMBINATION				
	Radiotherapy with cemiplimab	laCSCC	II	NCT05574101
	Anti EGFR antibody-dye conjugate, ASP-1929 with cemiplimab followed by photoimmunotherapy	laCSCC, mCSCC	I/II	NCT04305795
	OR502 (fully human IgG1 antibody that binds specifically to LILRB2) monotherapy and in combination with cemiplimab	laCSCC, mCSCC	I/II	NCT06090266
	SAR444245 (pegylated recombinant non-alpha IL2) in combination with cemiplimab	laCSCC, mCSCC	I/II	NCT04913220
	MK-3475A (pembrolizumab formulated with MK-5180)	laCSCC, mCSCC	II	NCT06041802
	Cetuximab in combination with pembrolizumab	recurrent and metastatic head and neck CSCC	II	NCT03082534
	Personalized neoantigen peptide vaccine in combination with pembrolizumab	laCSCC or mCSCC	I	NCT05269381
	SOT101 monotherapy and in combination with pembrolizumab	laCSCC, mCSCC	I	NCT04234113
	SOT101 in combination with pembrolizumab	CSCC	II	NCT05256381
	SGN-B6A monotherapy and in combination with pembrolizumab	laCSCC, mCSCC	I	NCT04389632
	Vilobelimab (IFX-1, monoclonal anti-C5a antibody) monotherapy and in combination with pembrolizumab	laCSCC, mCSCC	II	NCT04812535
	Elimusertib (BAY1895344), stereotactic body radiation, and pembrolizumab combination therapy	recurrent head and neck CSCC	I	NCT04576091
	MDNA11, long-acting "beta-only" recombinant interleukin-2 alone and in combination with pembrolizumab	laCSCC, mCSCC	I/II	NCT05086692
	BCA101 (targets both EGFR with TGFβ) alone and in combination with pembrolizumab	laCSCC, mCSCC	I	NCT04429542
	Nivolumab monotherapy and in combination with relatlimab	laCSCC, mCSCC	II	NCT04204837
	Radiotherapy with atezolizumab	laCSCC, mCSCC	I	NCT05085496
	NT-I7 (rhIL-7-hyFc) in combination with atezolizumab	laCSCC, mCSCC	I/II	NCT03901573
	Cobimetinib in combination with atezolizumab	laCSCC, mCSCC	II	NCT03108131
	Carrilizumab in combination with albumin-binding paclitaxel	laCSCC, mCSCC		NCT05886140
	Camrelizumab in combination with cisplatin	laCSCC, mCSCC	II	NCT05490485
	Avelumab monotherapy and in combination with cetuximab	laCSCC, mCSCC	II	NCT03944941
	GZ17-6.02 monotherapy and in combination with capecitabine	Advanced CSCC	I/II	NCT03775525
INTRATUMORAL/INTRALESIONAL				
	RP1 monotherapy and in combination with cemiplimab	laCSCC, mCSCC	II	NCT04050436
	Intratumoral Vidutolimod (CMP-001) in combination with cemiplimab	laCSCC or mCSCC	II	NCT04916002
	MQ710, a Multi-Transgene Expressing Modified Vaccinia Virus Ankara-Based Virotherapy, monotherapy and in combination with pembrolizumab	CSCC that have failed standard therapy	I	NCT05859074
	Intratumoral or intravenous TBio-6517, monotherapy and in combination with pembrolizumab	laCSCC or mCSCC that has not received systemic therapy	I/II	NCT04301011
	TransCon TLR7/8 agonist, monotherapy and in combination with pembrolizumab	laCSCC, mCSCC	I/II	NCT04799054
	Talimogene Laherparepvec and Nivolumab	Advanced CSCC	II	NCT02978625
	Talimogene Laherparepvec and Panitumumab	laCSCC, mCSCC	I	NCT04163952
	Microneedle Array Plus Doxorubicin	resectable stage I-III CSCC	II	NCT05377905
NEOADJUVANT				
	Cemiplimab	stage II to IV (M0) CSCC	II	NCT04154943
	Cemiplimab	stage III to stage IV (M0) CSCC of the head/neck, extremity, or trunk, and selected patients with stage II CSCC	I	NCT04975152
	Cemiplimab	stage II-IV CSCC of the head and neck	II	NCT03565783
	Cemiplimab	High-risk localized, regionally advanced or locally recurrent CSCC	II	NCT04315701
	Pembrolizumab	Stage II-IV laCSCC	II	NCT05025813
	Nivolumab or Nivolumab combined with Ipilimumab	resectable stage III-IVa CSCC	II	NCT04620200
	Atezolizumab	advanced CSCC	II	NCT04710498
	Cetuximab	Advanced CSCC		NCT02324608
	Erlotinib	aggressive resectable CSCC	I	NCT00954226
NEOADJUVANT + ADJUVANT				
	Cemiplimab	High risk CSCC	I	NCT04428671
	Cemiplimab	Resectable stage III cutaneous SCC	II	NCT04632433
	Pembrolizumab	PD-1 naïve high-risk resectable CSCC	II	NCT04808999
	Coformulated favezelimab/pembrolizumab (MK-4280A) or pembrolizumab	stage II to stage IV resectable laCSCC	II	NCT06036836
ADJUVANT				
	Cemiplimab	Following surgery and radiation therapy for high risk CSCC	III	NCT03969004
	Pembrolizumab	Following Surgery and Radiation for high risk laCSCC	III	NCT03833167
TRANSPLANT				
	Combination of nivolumab and ipilimumab with sirolimus and prednisone	Kidney transplant recipients with laCSCC or mCSCC	I/II	NCT05896839
	Ruxolitinib	Solid organ transplant recipients with mCSCC	II	NCT04807777
	RP1 intra-tumoral injection	laCSCC, mCSCC in solid organ transplant recipients or hematopoietic cell transplant	I/II	NCT04349436

### Adjuvant Use

Two trials designed to evaluate adjuvant pembrolizumab (NCT03833167) and cemiplimab (NCT03969004) are underway. In addition, several of the neoadjuvant trials mentioned above include adjuvant use as well. Based on the study designs, it is unclear whether adjuvant therapy is necessary if neoadjuvant therapy is given.

### Emerging Therapies

Although survival has improved following approval of immunotherapy, there are many patients with advanced CSCC who do not respond or develop resistance [31–33]. A number of strategies for overcoming immunotherapy resistance are being evaluated, including combination therapies [31–34]. Table 1 summarizes current ongoing trials for the treatment of advanced CSCC.

NCT06090266 is a trial assessing OR502 (IgG antibody that binds to LILRB2) either as monotherapy or in combination with cemiplimab. Pegasus Skin (NCT04913220) is currently investigating SAR444245 (THOR-707, pegylated recombinant IL-2) in combination with cemiplimab. NCT04305795 is a trial assessing combination cemiplimab with ASP-1929 (cetuximab and a light-activated dye) followed by photoimmunotherapy for CSCC. ASP-1929 with pembrolizumab is also being studied for head and neck SCC (HNSCC) with preliminary data showing an ORR of 29.4% [35]. RAMPART (NCT05574101) is a trial evaluating concurrent radiotherapy with cemiplimab. Additionally, a phase 2/3 trial (NCT06295809) evaluating whether personalized neoantigen therapy with novel mRNA vaccine V940 (mRNA-4157) can improve event-free survival in the neoadjuvant/adjuvant setting in combination with pembrolizumab compared to standard of care and pembrolizumab in CSCC has shown promising results in resected high-risk melanoma [36]. NCT06041802 is a trial studying MK-3475A, combination pembrolizumab and MK-5180, a small molecule inhibitor against aurora-A kinase [37]. NCT03082534 is investigating pembrolizumab with cetuximab for HNSCC with preliminary data showing an ORR of 45% at 6 months, exceeding published response rates for pembrolizumab or cetuximab monotherapy in this cohort [34]. NCT05269381 is a phase 1 trial investigating a vaccine targeting neoantigens on tumor cells in combination with pembrolizumab. AURELIO-04 (NCT05256381) is a trial studying SOT101, a subcutaneous IL-15Rβγ agonist in combination with pembrolizumab after phase 1 preliminary data (NCT04234113) showed encouraging results. NCT04389632 is a trial assessing SGN-B6A, an integrin beta-6-targeted antibody–drug conjugate as monotherapy and in combination with pembrolizumab. NCT04812535 is studying vilobelimab (IFX-1), a monoclonal anti-C5a antibody, alone and in combination with pembrolizumab. Vilobelimab has previously been studied in patients with severe sepsis or septic shock given C5a’s role in complement activation [38]. NCT04429542 is studying BCA101, a tumor-targeted bifunctional fusion antibody that simultaneously inhibits EGFR and TGFβ signaling as monotherapy and in combination with pembrolizumab. ABILITY-1 (NCT05086692) is evaluating MDNA11 (long-acting "beta-only" recombinant IL-2) alone and in combination with pembrolizumab. NCT04576091 is investigating combination therapy with elimusertib, a selective oral inhibitor of the Ataxia telangiectasia and Rad3 related kinase, stereotactic body radiation, and pembrolizumab for HNSCC.

Cosibelimab is a monoclonal antibody that binds to PD-L1 with a functional domain capable of inducing antibody-dependent and complement-dependent cytotoxicity against tumor cells [39]. An ORR of 47.4% was reported with a median time to response of 1.9 months and median follow-up of 15.4 months and lower rates of irAEs [39]. Only 23.1% of participants experienced irAEs with 2.5% experiencing grade 3 AEs. In July 2023, long-term data was reported with ORR of 55% for laCSCC (23% CR, 32% PR) and 50% for mCSCC (13% CR, 37% PR) [40]. Given positive results, an application was submitted to the FDA in January 2023 for approval of Cosibelimab for patients with advanced CSCC, however as of December 2023 the medication is not yet approved [41].

The trial arm of NIVOSQUACS (NCT04204837) studying nivolumab combination therapy with relatlimab is ongoing [27]. Relatlimab is a lymphocyte activation gene-3 inhibitor and combination nivolumab-relatlimab is being studied for HNSCC (NCT04326257). Atezolizumab, a monoclonal antibody against PD-L1, is being investigated as combination therapy for CSCC in several ongoing trials: NCT05085496 ( with radiotherapy), NCT03108131 (with cobimetinib), and NCT03901573 (with NT-17[efineptakin alfa], a long-acting IL-7 agonist).

Carrilizumab and camrelizumab are PD-1 inhibitors being studied in combination with chemotherapy: carrilizumab with paclitaxel (NCT05886140) and camrelizumab with cisplatin. NCT03944941 is a trial studying avelumab (a PD-L1 inhibitor) monotherapy and in combination with cetuximab. NCT03775525 is a trial of oral GZ17-6.02 (a combination of isovanillin, curcumin, and harmine) monotherapy and in combination with capecitabine. Afatinib is a tyrosine kinase inhibitor being studied for advanced CSCC (NCT05070403).

Several trials are evaluating oral PD-L1 inhibitors for CSCC (NCT04242199, NCT05888844, NCT04272034). HLX07, a recombinant anti-EGFR monoclonal antibody, is being assessed with preliminary results demonstrating an ORR of 23.8% at 9.6 months and 62.5% at 3.1 months depending on dosage [42].

Talimogene laherparepvec (TVEC) is an oncolytic viral therapy that is FDA approved for advanced melanoma. It has shown success for CSCC and has been explored in several clinical trials (NCT03714828, NCT03458117). T-VEC is also being explored in combination with nivolumab (NCT02978625) for advanced CSCC. Oncolytic viruses have been shown to improve antitumor immune response via multiple mechanisms and are being increasingly studied in clinical trials [43]. Beyond TVEC, a number of oncolytic viruses are currently being investigated both alone and in combination with anti-PD-1 therapies. The CERPASS trial (NCT04050436) is investigating combination cemiplimab and RP1, an oncolytic virus (HSV-1) that expresses a fusogenic glycoprotein and granulocyte macrophage colony stimulating factor. RP1 has shown success in combination with nivolumab for NMSC, including CSCC, in the IGNYTE trial (NCT03767348). NCT05859074 is evaluating MQ710, a multi-transgene expressing modified vaccinia virus, alone and in combination with pembrolizumab. NCT04301011 is investigating intratumoral and intravenous TBio-6517 (an engineered vaccinia virus that expresses the Flt3 ligand, an antibody targeting CTLA4, and IL-12) alone and in combination with pembrolizumab.

Intratumoral vidutolimod is a CpG-A TLR9 agonist that is currently being investigated in combination with cemiplimab (NCT04916002). Finally, a trial assessing intratumoral TransCon TLR7/8 agonist, a sustained-release prodrug of resiquimod, alone and in combination with pembrolizumab is ongoing (NCT04799054).

## Basal Cell Carcinoma

Basal cell carcinoma is the most common skin cancer with more than 3.6 million cases diagnosed annually [44]. It is highly correlated to UV exposure and skin type [1, 45, 46]. It is more common in older adults due to cumulative UV exposure but has been increasing in incidence for younger adults as well, which may be partially explained by artificial UV sources [47]. The cause of basal cell carcinoma is complex and multifactorial, including genetics (such as mutations in the Hedgehog signaling pathway), phenotype (lighter skin color), and environment (UV exposure).

For laBCC not amenable to curative intent local intervention, the current standard of care is initial treatment with a hedgehog inhibitor (HHI) (either vismodegib or sonidegib). For mBCC, vismodegib is the initial HHI of choice. Alternative dosing schedules or drug holidays can be used for intolerable side effects. For patients who were previously treated with a HHI or who cannot tolerate an HHI, cemiplimab is the standard second-line therapy.

### Hedgehog Inhibitors

70% of sporadic BCCs have PTCH1 (protein patched homolog 1) mutations, the receptor for Sonic Hedgehog (Shh) [48]. When Shh binds PTCH1, smoothened (SMO), an associated protein suppressed by unbound PTCH1, loses this inhibition and becomes activated, leading to cell proliferation [49]. Inactivating mutations in PTCH1 or activating mutations in SMO are both commonly found in BCC. Indeed, inherited mutations in PTCH1 lead to Gorlin Syndrome (nevoid basal-cell carcinoma syndrome), a condition associated with development of multiple BCCs, particularly at a young age [50]. Attempts to target the PTCH1/Shh/SMO pathway led to the development of Hedgehog pathway inhibitors [51].

Vismodegib was the first oral SMO inhibitor developed to target this pathway. It was FDA approved in January 2012 for treatment of locally advanced (laBCC) or metastatic BCC (mBCC) based on results of the ERIVANCE BCC trial [52]. In this trial, ORR was 30% in patients with mBCC and 43% in patients with laBCC. The median progression free survival (PFS) for mBCC was 9.2 months and 9.5 months for laBCC. 25% of patients developed grade III-IV adverse effects, with muscle spasms, alopecia, weight loss, and dysgeusia being the most common. Meta-analyses have found similar results with an average ORR of 64.7% for laBCC and 33.6% for mBCC. Patients were on vismodegib for an average of 35.8 weeks [53]. In this analysis, 28.2% of patients discontinued vismodegib due to adverse effects, with muscle spasms, dysgeusia, and alopecia being the most common effects noted.

Due to the durable effects of vismodegib in cases where surgical resection was not amenable, vismodegib was studied in the neoadjuvant setting in patients with laBCC with the goal of tumor shrinkage prior to surgical resection. Large tumor size may render certain patients inoperable or have significant morbidities associated with resection such as functional deficits or disfiguration. In the VISMONEO trial, 80% of patients with locally advanced BCC who received neoadjuvant vismodegib achieved downstaging of surgical resection after a mean treatment duration of 6 months [54]. 36% of patients had known recurrence at 3 years of follow-up. There was a 20% incidence of grade III-IV adverse effects. Neoadjuvant use of vismodegib has also been studied in the use of periocular laBCC where it had an ORR of 75% and reduced rates of orbital exenteration (surgical removal of the eyeball and surrounding tissues) from 46 to 6% [55, 56].

Sonidegib is another oral SMO inhibitor that is currently approved for laBCC. In the BOLT trial, the ORR (while on 200mg) for mBCC was 8% and 56% for laBCC. Median PFS was 13.1 months for mBCC and 22.1 months for laBCC. There was a 43% incidence of grade 3–4 adverse effects with a side effect profile comparable to vismodegib. There have been no head-to-head trials comparing vismodegib to sonidegib [57]. In an indirect comparison, sonidegib had a higher ORR and PFS compared to vismodegib with slightly lower rates of adverse effects [58, 59].

Adverse effects are a limiting factor in the long-term tolerability of HHIs for patients. In addition, these adverse effects are class-specific, meaning that sonidegib and vismodegib will be tolerated similarly for a specific patient and switching agents due to adverse effects will likely not lead to better tolerability [60]. As a result, altering the drug-regimen schedule in response to adverse effects has become protocolized with similar efficacy as seen in the original clinical trials [61, 62].

Tadadegib is a new HHI currently under investigation. Phase I data showed an appropriate safety profile and clinical response but there are not currently any active phase II trials [63]. Clinical trials of topical hedgehog inhibitors (patidegib) in order to minimize systemic absorption and adverse effects are currently under investigation [64]. Interestingly, itraconazole, an antifungal medication, is a strong antagonist of the Hedgehog inhibitor pathway, in a mechanism different from oral SMO inhibitors, and is also being studied in the treatment of BCC [65].

Patients on HHIs can develop resistance during treatment, leading to refractory disease. Analyses have shown that most cases of HHI resistance are due to mutations of SMO that lead to alterations in how the drug accesses its binding site or mutations in the binding site itself. There are also downstream targets that have been found to have mutations, conferring resistance [66, 67].

### Immunotherapy

Cemiplimab was approved in February 2021 for treatment of laBCC in patients who already progressed through or were intolerant to HHIs. In long-term follow-up of NCT03132636, there was an ORR of 32.1% with a median PFS of 15.6 months. 48% of patients suffered grade III-IV adverse events [68, 69]. There have been case series of other immunotherapy agents, such as pembrolizumab, given with and without oral SMO agents with similar effectiveness (44% ORR for pembrolizumab alone and 29% for combination therapy) [70].

## Merkel Cell Carcinoma

Merkel cell carcinoma is a rare and aggressive skin cancer that has neuroendocrine features. It is neuroendocrine in origin due to immunohistochemistry revealing chromogranin A, synaptophysin, and CD56, as well as somatostatin receptors [71, 72]. Despite the tumor cell’s resemblance to Merkel cells which are mechanoreceptors in the dermis of the skin, it is currently believed that MCC does not arise from Merkel cells and the true origin has yet to be elucidated [73]. 80% of cases of MCC are thought to be due to the Merkel cell polyomavirus (MCV) and cases not associated with MCV are thought to be due to DNA damage from UV radiation [74, 75].

For mMCC, standard therapy includes anti PD-1 therapy (avelumab, pembrolizumab, nivolumab, or retifanlimab). Avelumab or pembrolizumab are most used due to their prevalence.

### Cytotoxic Chemotherapy

Before the advent of immunotherapy, cytotoxic chemotherapy was used as first-line for patients with metastatic MCC (mMCC). While multiple different regimens were used, the most common were etoposide with either carboplatin or cisplatin [76]. 55% of patients responded to first-line chemotherapy and 23% of patients responded to second-line therapy with a median overall survival of 9.5 months from the start of chemotherapy.

Cytotoxic chemotherapy is still used for patients who have progressed through or who cannot tolerate immunotherapy and is also used for palliative debulking and symptomatic management [77]. There have been studies investigating if adjuvant use of systemic chemotherapy after surgery or radiation improves survival and, so far, no survival benefit has been seen [78, 79].

### Immunotherapy

PD-L1 expression was found in the MCC microenvironment in 49% of tumor cells, making it a possible target in the treatment of mMCC [80]. Avelumab (PD-L1 inhibitor) became the first FDA-approved agent for the treatment of mMCC in 2017 [81]. Evidence for this use came out of the results of the JAVELIN Merkel 200 trial which showed ORR of 33% and median PFS of 2.7 months in patients with mMCC who had progressed on cytotoxic chemotherapy [82, 83]. Only 10% of patients had grade III-IV adverse effects. There was a trend towards a lower ORR for patients with MCV-positive versus MCV-negative tumors (26% versus 46%) but the results were not statistically significant. In comparison, patients with mMCC who were chemotherapy-naïve were shown to have a higher ORR (56.3% versus 33%) [84].

Pembrolizumab was approved for mMCC in 2018. In the KEYNOTE-017 trial, patients with mMCC who were chemotherapy-naïve had an ORR of 56% with a median PFS of 16.8 months [85]. The response rate was higher among those with MCV-positive tumors than those with MCV-negative tumors (62% versus 44%). 28% of patients had grade III-IV adverse effects. An extended analysis with a median follow-up of 31.8 months showed an overall response rate of 58% [86]. There have been no head-to-head comparisons of avelumab versus pembrolizumab.

Retifanlimab is another PD-1 inhibitor approved for advanced or metastatic MCC. The POD1UM-201 trial showed an ORR of 46.2% among chemotherapy-naïve patients with 28.7% of patients having grade III-IV adverse effects [87]. Nivolumab has been studied in the neoadjuvant setting for MCC. In the CheckMate 358 trial, 47% of patients with resectable MCC who received neoadjuvant nivolumab achieved a pathologic complete response after surgery and 54.5% had tumor reduction of 30% or greater with a median follow-up of 26 weeks [88]. 7.7% of patients had grade III-IV adverse effects. Given the availability of durable responses both avelumab and pembrolizumab have shown and limited long-term data with retifanlimab or nivolumab, these two agents are not commonly used.

Combination immune-checkpoint inhibitors (ipilimumab and nivolumab) have also been studied for advanced MCC. Patients who were immunotherapy-naïve had an ORR of 100% while patients who had been previously treated with immunotherapy had an overall response rate of 31%, with higher toxicity profiles than patients on single immunotherapy agents alone [89] In another study looking at combination therapy for patients who progressed on single agents, 31% of patients experienced a grade III-IV immune-related adverse event with a median overall survival of 4.7 months from the initiation of combination therapy [90]. As a result of a high toxicity profile and conflicting data on whether or not combination therapy provides improved responses to single therapy alone, combination therapy is only recommended for use in patients who have progressed on single therapy [89–93].

Adjuvant nivolumab has been studied after complete resection of MCC in the ADMEC-O trial. Nivolumab was found to have an absolute risk reduction of 10% of disease-free survival versus observation (84% versus 73%) with 42% of patients treated with nivolumab having grade 3–4 adverse events versus 11% of patients under observation [94].

### Emerging Therapies

Like other neuroendocrine tumors, MCC expresses somatostatin receptors, activation of which generally triggers inhibitory pathways downstream. The use of somatostatin analogues in the treatment of mMCC has been studied in case series with some success [95, 96]. Currently, there are clinical trials ongoing looking at the use of peptide receptor radionuclide therapy in combination with immunotherapy for mMCC, which has already shown success in small case series [97, 98].

T-VEC has shown some success in case reports when used for advanced MCC [99, 100]. It is currently being investigated in clinical trials [101].

Many other clinical trials are ongoing. The MERCURY trial is looking at neoadjuvant retifanlimab plus platinum-etoposide therapy for resectable MCC (NCT05594290) [102]. The I-MAT trial is a phase II trial investigating adjuvant avelumab in stage I-III MCC (NCT04291885) [103] The ADAM trial is a phase III trial investigating adjuvant avelumab in stage III MCC (NCT03271372) [104]. The KEYNOTE-913 trial is a phase III study looking at pembrolizumab in untreated advanced MCC (NCT03783078) [105]. The MERKLIN2 trial is a phase II study investigating domatinostat, a histone deacetylase inhibitor, in combination with avelumab in patients with advanced or metastatic MCC who have progressed on anti-PD-L1 therapy (NCT04393753) [106]. NCT03787602 is a study investigating KRT-232, a MDM2 inhibitor, for MCC in patients who failed treatment with anti-PD-1 or anti-PD-L1 therapy [107].

## Transplant Patients

Immunocompromised patients have a higher risk of developing CSCC with studies suggesting poorer outcomes [108]. It has been shown that cost of skin-cancer related dermatological care for transplant recipients is higher when compared with non-immunosuppressed patients, which is primarily driven by CSCCs in transplant patients requiring advanced and/or multimodal treatment regimens [109].

Among solid organ transplant recipients (SOTRs), risk of rejection is notable with one study reporting it to be as high as 37% [108]. Several clinical trials are exploring safety and efficacy of immunotherapy for SOTR with advanced skin cancer. CONTRAC-1 (NCT04339062) reported results of a phase I study of cemiplimab for renal transplant recipients with advanced CSCC [110]. Twelve patients received cemiplimab with a standardized approach to immunosuppression with mTOR inhibition and dynamic prednisone. Of 11 evaluable patients, 5 responded at median follow-up of 6.8 months (3 CR and 2 PR), no patients experienced rejection or graft loss [111]. Two patient had stable disease. One patient died of angioedema and anaphylaxis which was attributed to the mTOR inhibitor [110]. NCT03816332 is a phase I/II trial assessing combination nivolumab plus tacrolimus plus prednisone ± ipilimumab in renal transplant patients with advanced skin cancers, including 5 patients with CSCC and 2 with MCC [112]. All patients had progressive disease with nivolumab + tacrolimus + prednisone. For those with CSCC, 3 patients received nivolumab + tacrolimus + prednisone + ipilimumab resulting in 2 CR and 1 stable disease. One of the patients with CR experienced treatment-related allograft loss 6 weeks after starting nivolumab. Both patients with MCC were subsequently treated with addition of ipilimumab however both had progressive disease and one experienced allograft loss 8 weeks after discontinuation of therapy [112]. Thus, the risk of rejection must be clearly discussed with transplant patients being considered for immunotherapy and patients should be evaluated on a case-by-case basis pending future studies. Immunosuppression with mTOR inhibition and a pulsed steroid approach for transplant patients on immunotherapy may be considered.

## Conclusion

In this review, we examined the latest advances of systemic therapies for advanced and metastatic non-melanoma skin cancers. Understanding the pathophysiology and molecular background of NMSC has become increasingly essential as more and more targeted therapies are being developed and studied. Immunotherapy has greatly expanded treatment options across all types of NMSC and its use in both neoadjuvant and adjuvant settings are actively being investigated. As the incidence of non-melanoma skin cancer continues to rise, clinicians should feel better equipped to treat advanced and metastatic NMSC.

## Data Availability

No datasets were generated or analysed during the current study.
